# Distribution of cortactin in cerebellar Purkinje cell spines

**DOI:** 10.1038/s41598-020-80469-w

**Published:** 2021-01-14

**Authors:** Lilla E. Szabó, G. Mark Marcello, Miklós Süth, Péter Sótonyi, Bence Rácz

**Affiliations:** Department of Anatomy and Histology, University of Veterinary Medicine Budapest, István u. 2., 1078 Budapest, Hungary

**Keywords:** Cellular neuroscience, Neuroscience

## Abstract

Dendritic spines are the primary sites of excitatory transmission in the mammalian brain. Spines of cerebellar Purkinje Cells (PCs) are plastic, but they differ from forebrain spines in a number of important respects, and the mechanisms of spine plasticity differ between forebrain and cerebellum. Our previous studies indicate that in hippocampal spines cortactin—a protein that stabilizes actin branch points—resides in the spine core, avoiding the spine shell. To see whether the distribution of cortactin differs in PC spines, we examined its subcellular organization using quantitative preembedding immunoelectron microscopy. We found that cortactin was enriched in the spine shell, associated with the non-synaptic membrane, and was also situated within the postsynaptic density (PSD). This previously unrecognized distribution of cortactin within PC spines may underlie structural and functional differences in excitatory spine synapses between forebrain, and cerebellum.

## Introduction

Glutamatergic axospinous synapses are the most abundant type of excitatory neuronal connection in the mammalian brain ^1^. Activity-dependent long-term synaptic plasticity at axospinous synapses is thought to underlie associative learning ^2^. Our understanding of the mechanisms underlying mammalian synaptic plasticity is largely based on studies of forebrain neurons, especially in the hippocampus, but there is reason to suspect that principles derived from this research may not apply to the cerebellum ^3^. The cerebellum is a phylogenetically-conserved brain structure that develops from the anterior hindbrain ^4^. The cerebellar cortex contains tiny excitatory granule cells (GCs) densely packed in the granule cell layer (GCL), representing nearly 80% of the total number of neurons within the brain. Purkinje cells (PCs) are giant inhibitory neurons, whose somata are restricted to a very thin layer (Purkinje cell layer, PCL); their dendrites branch extensively in the overlying molecular layer. The fundamental circuitry of the cerebellum is reliant on these two cell types. Each GC receives its excitatory afferent input from a few mossy fibers (MFs), which originate from outside the cerebellum. GCs then emit a single parallel fiber (PF), which runs in the molecular layer and makes axo-spinous contacts with thousands of PCs. Each PC receives input from hundreds of thousands of PFs^5^. The activity of PCs is also modified by several hundred axodendritic synapses from a single climbing fiber (CF) whose cell body lies in the inferior olivary nucleus. PCs provide the exclusive output of the cerebellar cortex.

The physiological basis of long-term synaptic plasticity in these two brain regions is quite different. Long-term potentiation (LTP), thought to underlie associative learning in the forebrain ^6^, is NMDAR dependent. However, postsynaptic densities (PSDs) of PCs possess very few NMDARs, mainly restricted to CF-PC synapses ^7,8^. Instead, PC spines express high levels of mGluR1 ^9^. Cerebellar learning arises from the temporal pairing of signals from MFs onto PC spines with activation of the associated CF, leading to LTD at the PF-PC synapse that is mechanistically quite different from forebrain synaptic plasticity. Interestingly, the PF-PC synapse can also express LTP postsynaptically, but the induction of this cerebellar LTP relies on protein phosphatases not on kinases—just the opposite of synaptic plasticity requirements observed in forebrain synapses ^10–15^. Nevertheless, according to the currently accepted view, LTD and LTP play a complementary role in cerebellar motor learning.

These long-lasting changes in synaptic efficacy are tightly coupled to changes in spine morphology, at least in the forebrain ^16^. Sophisticated protein machinery inside the spine coordinates the reorganization of the actin cytoskeleton necessary for synaptic ‘morphing’ ^17,18^. Numerous proteins have been implicated in spine remodeling, pointing to an intricate network of pathways that regulate actin dynamics. Accumulating evidence for spatial restriction of these actin-binding proteins suggests that molecular compartmentalization provides a structural basis for regulation of the spinoskeleton ^19^.

One such protein, cortactin, has a central actin-binding domain, and its N-terminal interacts with the Arp2/3 complex ^20–22^. This interaction supports the stabilization of actin filaments and promotes their branching. Cortactin is implicated in the dynamic reorganization of dendritic spines ^23,24^. Previous studies have shown that cortactin in hippocampal pyramidal cells concentrates in dendritic spines, where it resides in the spine core ^23,25^. Purkinje cells also express cortactin ^26^. One might presume a similar organization of cortactin in PCs as in the cerebellar cortex, but in light of the dramatic developmental and functional differences between cerebellum, and hippocampus, this hypothesis is speculative. Therefore, we performed quantitative immuno-electron microscopy to localize cortactin within PC spines, aiming to elucidate a possible molecular basis for the functional differences between forebrain and cerebellar spines.

## Materials and methods

Experiments were performed on Wistar rats from Toxi-coop (Budapest, Hungary). Animal housing, all experimental procedures, and protocols were approved by the Institutional Animal Care and Use Committee (Animal Welfare Committee of the University of Veterinary Medicine Budapest—permission number: #MAB53/2013). Our experiments were strictly in compliance with guidelines, and relevant laws, and the study was carried out in compliance with the ARRIVE guidelines (http://www.nc3rs.org.uk/page.asp?id=1357). Animals were maintained in standard laboratory conditions, at 22 °C, with 12 h light–dark cycles, on Lignocel bedding (Toxi-coop, Budapest). We deeply anesthetized 3 month old male rats (weight 250–300 g) with pentobarbital (60 mg/kg, i. p.; Eutanyl, Bimeda MTC, ON, Canada), and perfused transcardially with 0.9% NaCl, then fixed with a mixture of depolymerized paraformaldehyde (PFA, 4%, Sigma, Germany), and 0.2% glutaraldehyde (Electron Microscopy Sciences, PA, USA) dissolved in 0.1 M phosphate buffer (PB), pH = 7.4. Brains were removed, and postfixed overnight at 4 °C in 4% PFA dissolved in 0.1 M PB. 60 μm midsagittal plane sections were cut from the cerebellar hemispheres with a Leica vibratome (VT1000, Leica, Wetzlar, Germany), and processed for immunoelectron microscopy. We took samples for our study from the outer molecular layer of lobules VI, and VII of the cerebellar cortex, approx. − 15 mm caudal from Bregma, and + 0.5–0.9 mm mediolaterally (sagittal plane), in the vicinity of the pre-pyramidal and post-superior fissures^25,27–29^.

### Antibodies

The following widely utilized and well-characterized primary antibodies were used in this study: polyclonal rabbit anti-cortactin (0.4 μg/ml; Santa Cruz Biotechnology, Santa Cruz, CA, USA). This antibody was used by ^30^ and ^25^. We verified its specificity with Western blot analysis, finding that the antibody recognized two bands in the ∼80–85 kDa range in the cortex, hippocampus, and cerebellum ^25^. We also used polyclonal chicken anti-Calbindin D28k (#214 006, 1:5000, Synaptic Systems, Goettingen, Germany), monoclonal mouse anti-synaptophysin (clone SVP38, 1:1000; Sigma, St. Louis, MO, USA; see ^31^ and ^32^), and monoclonal mouse anti-PSD-95 (#810401, 1:500 BioLegend, San Diego, CA, USA). Sections were incubated overnight with primary antibodies ^25,28,29^.

### Immunocytochemistry for confocal microscopy ^25,28,29^

Floating sections were blocked in 0.1 M phosphate buffered saline (PBS), complemented with 20% normal donkey serum (NDS; Jackson ImmunoResearch, West Grove, PA, USA) at pH 7.4, then incubated with primary antibodies for cortactin and synaptophysin in 0.1 M 2% NDS/PBS overnight at room temperature. This was followed by washes, and incubation in secondary antibodies (anti-rabbit-Alexa-488 for cortactin, anti-mouse-Cy3 for synaptophysin). After this, sections were mounted on glass slides, coverslipped in Fluoroshield with DAPI (Sigma, Darmstadt, Germany), and examined with an SP2 laser scanning confocal microscope (Leica, Wetzlar, Germany). Optical sections were acquired with a Plan Apo 63 × oil objective (numerical aperture 1.4) and were scanned in a 1024 × 1024 pixel format. Images stored as RGB TIFF images were digitally processed with Adobe Photoshop (version 7.0; Adobe Systems, Mountain View, CA, USA). Each image was cropped, and sharpened with unsharp masking; contrast, brightness, tonal range, and color balance were edited. Each processing step was applied uniformly to the entire image.

### Immunocytochemistry for electron microscopy^25,28,29^

To quench free aldehyde groups, sections were treated for 30 min in 1% sodium borohydride in 0.1 M PB; to suppress nonspecific binding, the sections were incubated in 20% NDS for 25 min followed by a 12 h incubation in anti-cortactin antibody, along with 2% NDS. After incubation in biotinylated anti-rabbit IgG (Jackson) for 2 h and washes in PBS, the sections were incubated in Extravidin peroxidase (1:5000; Sigma, Germany) for 1 h. Immunopositive structures were visualized with 3,3′-diaminobenzidine tetrahydrochloride (DAB; Merk, Germany). As control, the primary antibodies were omitted from the incubation solution; no immunostaining was observed in these cases. Some immunoperoxidase-stained sections were prepared for light microscopy; these were mounted on glass slides, dehydrated in ascending ethanol series, cleared with xylene, and coverslipped with DPX (Merck, Germany) mountant.

For preembedding immunogold labeling, sections were incubated together with those for immunoperoxidase staining up to the secondary antibody stage. In control section, the primary antibody was either omitted or) replaced with normal rabbit serum as a positive control (Fig. 5D, Jackson). After rinses in PBS, sections were incubated in biotinylated donkey-anti rabbit IgG (Jackson) for 30 min. After washes in 0.1 M PB, sections were incubated in 1.4 nm Nanogold-Streptavidin (1:100; Nanoprobes, Yaphank, NY, USA) for 1 h at room temperature and rinsed in PB. Sections were washed in 0.1 M Na acetate (to remove phosphate and chloride ions), followed by gold enhancement with GoldEnhance EM Plus (Nanoprobes, Yaphank, NY, USA) for ~ 8 min.

Sections for electron microscopy were postfixed with 0.5% OsO_4_ in 0.1 M PB for 30 min, as described earlier ^25,28,29^. After dehydration in ascending ethanol series, and contrasting with 1% uranyl acetate for 1 h in 70% EtOH, sections were incubated in propylene oxide, infiltrated with Durcupan resin (Sigma, Germany), and flat-mounted between sheets of Aclar (EMS, PA, USA) within glass slides. 70 nm sections were cut, mounted on 300 mesh copper grids, contrasted with lead citrate (Ultrostain II, Leica, Germany), and examined in a TEM-1011 (JEOL, Tokyo, Japan) electron microscope at 80 kV; images were collected with a Megaview 12-bit 1024 × 1024 CCD camera.

### Quantitative analysis of the immunogold reaction

As described in previous studies^25,28,29^, electron micrographs were taken from randomly selected fields ^26,30,31^. We focused on the distal molecular layer of the cerebellar cortex, extending out superficially at least ∼50–100 μm from the Purkinje cell layer. For details on measuring gold particle positions, see previous reports ^25,28,29,33^. Distances, membrane perimeters, and profile areas were measured using ImageJ version 1.52n software (National Institutes of Health, Bethesda, MD, USA). Microsoft Excel, and Kaleidagraph (Synergy Software, Reading, PA, USA) were used to generate graphs, and to compute statistics. Numberical data are presented as mean ± SEM.

### Identification of the PC-PF spine synapses

The overwhelming majority of axospinous synapses on PCs are from GC PFs ^5^. CFs may also establish spine-synapse type connections, but these are sparse and restricted to the initial proximal dendrites of PCs ^34^. In our sample (targeting the distal PC arbors) the vast majority of synapses were PC-PF contacts. GC terminals contacting PC spines can be distinguished based on their morphology: PF terminals contain small clusters of synaptic vesicles concentrating at the synaptic contact site, while CF boutons contain a high density of evenly distributed vesicles ^35^. Based on clear morphological clues, we could reliably identify and analyze a homogenous PF-PC spine population, and the very limited degree of possible misidentification is likely to have only marginal impact on our results.

## Results

**Figure 1:**
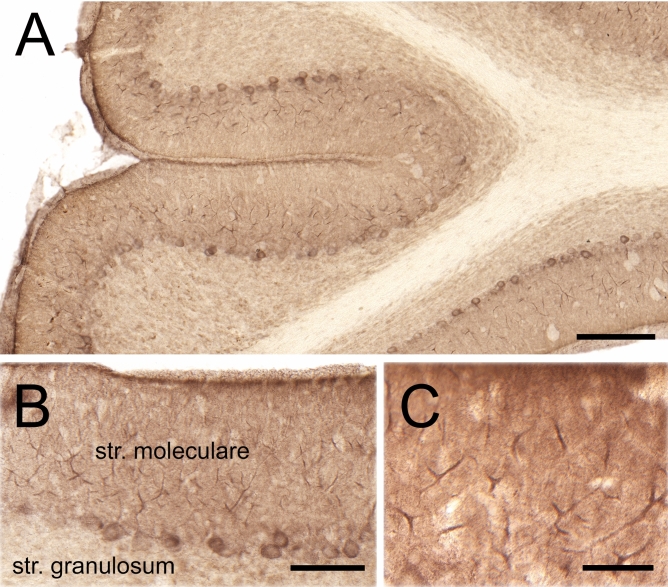
Immunoperoxidase staining for cortactin in cerebellum. **(A)** Low magnification view of cerebellar cortex. (**B)** The three layers of the cortex can be seen: inner layer: str. granulosum, Purkinje-cell layer in the middle, outer layer: str. moleculare. Purkinje cell bodies can be seen at the border between str. granulosum and str. moleculare. (**C)** Staining is conspicuous within the dendritic arbor of PCs in str.moleculare. Scale bars: **(A)**: 200 µm, **(B)**: 100 µm, **(C)**: 50 µm.

**Figure 2:**
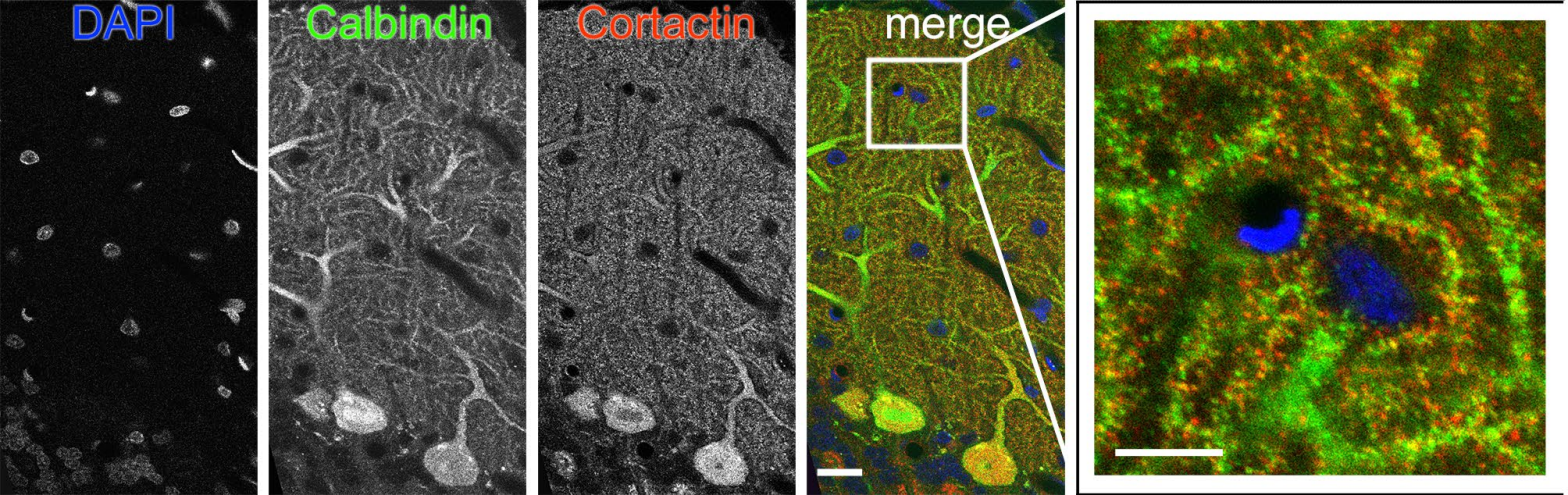
Cortactin colocalizes with calbindin in PCs. Confocal microscopy shows cell nuclei (DAPI, blue), the PC-specific calcium-binding protein Calbindin (Alexa-488, green), and cortactin (Cy3, red). The two markers almost completely colocalize in the molecular layer of the cerebellum. The inset is the expanded image of the region surrounded by a white rectangle. Scale bar: 20 µm, 10 µm for the inset.

**Figure 3:**
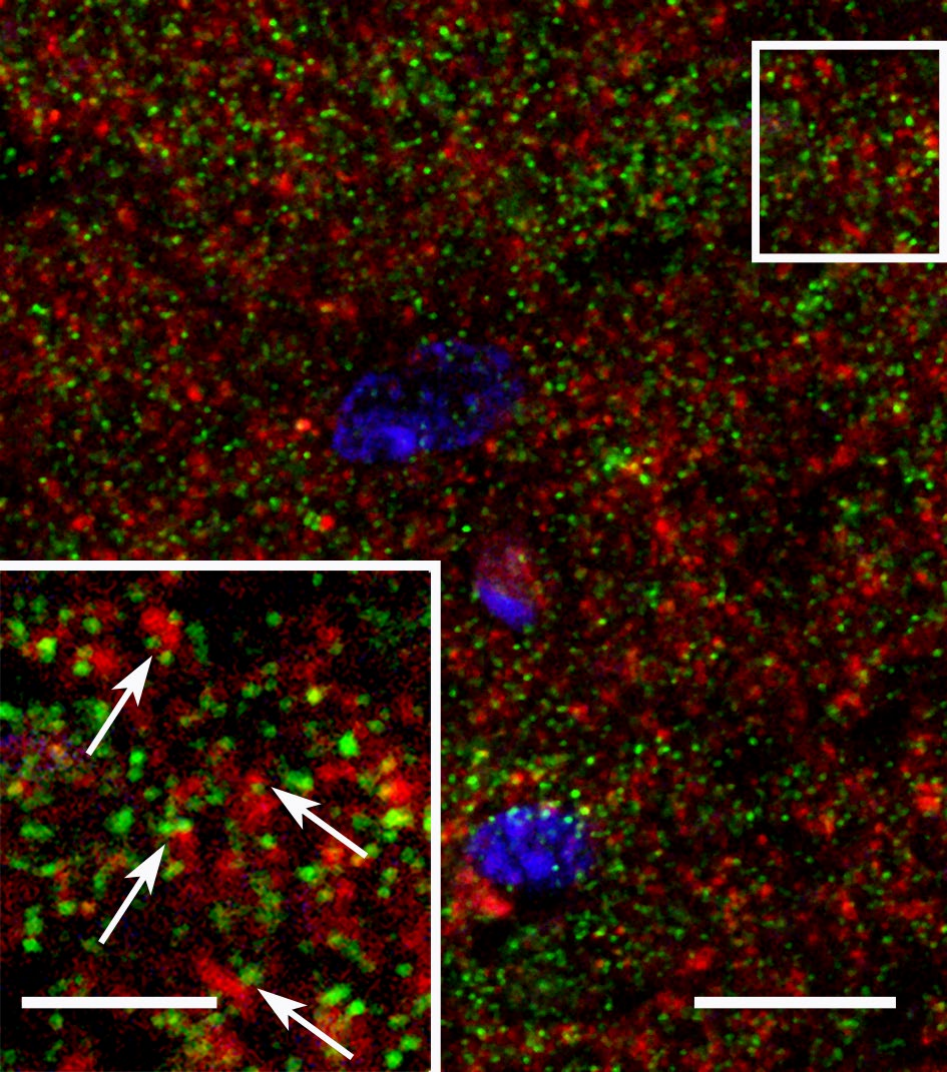
Subcellular distribution of cortactin in the outer molecular layer of the cerebellar cortex. Confocal microscopy shows cortactin (Cy3, red) and the presynaptic marker synaptophysin (Alexa-488, green); cell nuclei (DAPI) are shown in blue. The apposition of puncta immunopositive for cortactin and synaptophysin (arrows in inset) suggest that cortactin is likely localized to postsynaptic structures. The inset is an expanded image of the region surrounded by a white rectangle. Scale bar: 10 µm (5 µm for inset).

**Figure 4:**
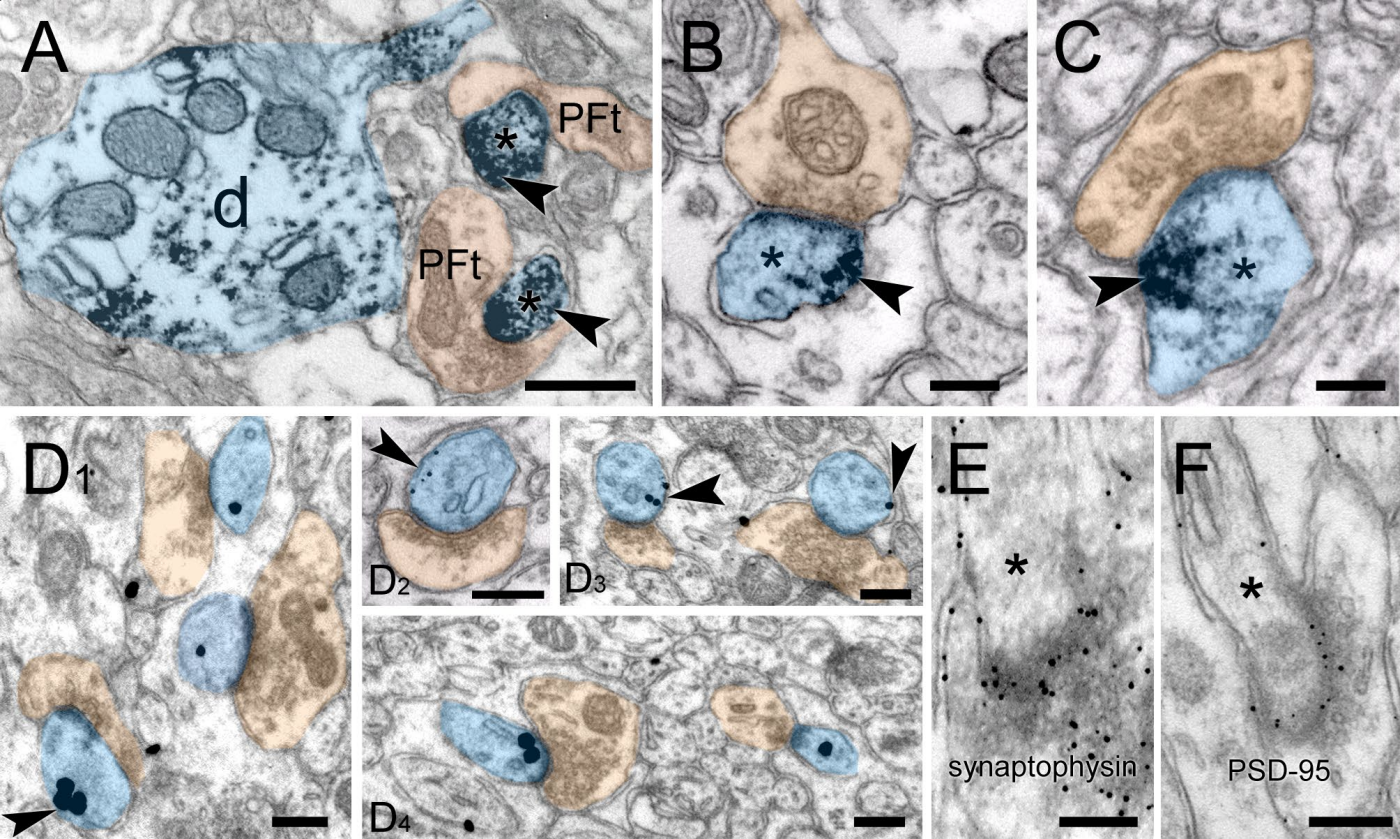
Immune-electronmicroscopy of cortactin in spines of distal PC branchlets. **(A–C)** Robust postsynaptic DAB reaction product (blue pseudo-color) in Purkinje-cell dendrites (d), and spines (*) (parallel fiber terminals (PFt) seen in orange pseudo-color). Arrowheads point to accumulated patches of immunoperoxidase reaction product at submembrane regions. (**D**_**1–4**_**)** Immunogold label coding for cortactin. Dendritic spines of PCs (blue pseudo-color); cortactin-coding immunogold particles may be seen in the periphery of spines associated with synaptic membranes, non-synaptic membranes, and submembrane spinoplasm (arrowheads). (**E,F)** Immunogold particles coding for synaptophysin (**E**) may be seen labelling presynaptic terminals. In contrast, PSD-95 (**F**) label is restricted to the postsynaptic density of PC spines. Scale bar: **(A)**: 500 nm, and **(B–F)**: 250 nm.

Cortactin immunostaining was widespread in the gray matter of cerebellar cortex, sparing white matter. Staining in the cerebellar cortex was dense in the str. moleculare (outer layer), where the dendrites of PCs arborize (Fig. 1A–C). To ensure that Purkinje neurons indeed express cortactin, we performed high-resolution laser-scanning confocal microscopy on cerebellar sections double labelled with the PC-specific marker calbindin (Fig. 2). We confirmed that PCs are indeed expressing cortactin in their dendritic tree, and in puncta associated with their dendritic branchlets, suggesting that spines are enriched in cortactin. To identify whether cortactin is primarily localized to postsynaptic dendritic spines, we performed additional confocal microscopy on cerebellar sections double labelled for cortactin and the presynaptic marker synaptophysin. Cortactin showed punctate staining in str. moleculare; these puncta were frequently associated with synaptophysin puncta (Fig. 3). There was seldom overlap between puncta stained for cortactin and puncta stained for synaptophysin, suggesting that cortactin in cerebellar PCs concentrates postsynaptically. Taken together, our light microscopy (LM) data show that cortactin is expressed at high levels in the str. moleculare of the cerebellar cortex, where it most likely concentrates in dendritic spines. To gain insight into cortactin’s subcellular compartmentalization we used immuno-electron microscopy. We performed immunoperoxidase labeling to see if PC spines were labeled for cortactin. Although occasional axon terminals, and glial end-feet were immunopositive, most of the reaction product was found in spines, where it was organized into patches (Fig. 4A–C, arrows). Reaction product was often restricted to submembrane areas of PC spines, and it was generally weak or absent from the central core of the spinoplasm. Reaction product was seldom detected in axon terminals. Thus, our immunoperoxidase labeling confirmed that cortactin tends to concentrate in postsynaptic PC spines.

Peroxidase labeling does not allow precise localization as the reaction product may migrate away from the enzymatic site, it often fills a given profile making precise quantitative localization of the antigen impossible. To circumvent these limitations of peroxidase labeling, we performed pre-embedding immunogold labeling followed by gold intensification, to provide quantifiable spatial localization of cortactin (Fig. 4). To be sure that the immunogold label was indeed predominantly accumulating in spines, as seen in our immunofluorescence, and immunoperoxidase experiments, we first assessed the density of gold particles in different subcellular compartments. Nonspecific background labeling was estimated by measuring gold particle density over nuclei of Purkinje cells, as these were not stained with LM techniques (Fig. 1). Labeling over axon terminals was not significantly different in comparison with the nuclear staining, however particle density was markedly elevated in dendrites and dendritic spines (~ 7 and ~ 25 times above background, respectively; Table 1). In addition, to demonstrate the consistency of labeling in PC spines and lack of labeling in PF terminals we followed labeled spine profiles in serial ultrathin sections (see Supplementary Fig. S1 online).

**Table 1:** Cortactin labeling gold particle density over various neuropil compartments in the superficial molecular layer) of the cerebellar cortex.

	Density (particles/µm^2^)
PC nuclei	0.44 ± 0.04 (n = 16)
Axon terminals (asymmetric synapses)	1.22 ± 0.28 (n = 99)
PC dendrites	3.4 ± 0.85 (n = 15)**
PC spines	12.28 ± 1.09 (n = 170)***

Random profiles were classified into the above categories, regardless of immungold content. To assess non-specific/background labeling, Purkinje cell nuclei (from the Purkinje cell layer) were also examined. Spines and shafts of PCs showed robustly significant differences from nuclear background (P < 0.001, and P < 0.01, respectively), while axon terminals had notably fewer particles, not significantly different from the background (P = 0.8). (± values: one-way ANOVA with post-hoc Tukey tests; n: profile sample size).

Using material from both immunoperoxidase- and immunogold-labeled neuropil, we measured major and minor axis length, area, and perimeter of presynaptic PF terminal profiles, and postsynaptic PC spine profiles (Table 2). We also measured the number of multi-synaptic boutons, finding that the overwhelming majority of PC spines contacted a single PF bouton (1.04 ± 0.02, see Table 2). As CF varicosities typically form multi-synaptic contacts with spines, these results provide further support that we sampled from a relatively homogenous PC-PF spine pool.

**Table 2:** Ultrastructural analysis of different morphological features of PC spines, and presynaptic terminals in the superficial outer layer (str. moleculare) of the cerebellar cortex.

	MA (nm ± SE)	ma (nm ± SE)	MA/ma ratio (± SE)	Area (µm^2^ ± SE)	Perimeter (nm ± SE)	Spines per terminal (± SE)
PC spines (n = 102)	547 ± 21	316 ± 11	1.88 ± 0.1	0.149 ± 0.0075	1506 ± 46	1.04 ± 0.02
PF terminals (n = 108)	628 ± 20	280 ± 15	2.77 ± 0.17	0.211 ± 0.0111	1967 ± 53	

Major axis length (MA), minor axis length (ma), ratio (calculated, MA/ma), area, perimeter, and multi synaptic bouton density (spines per terminal).

**Figure 5:**
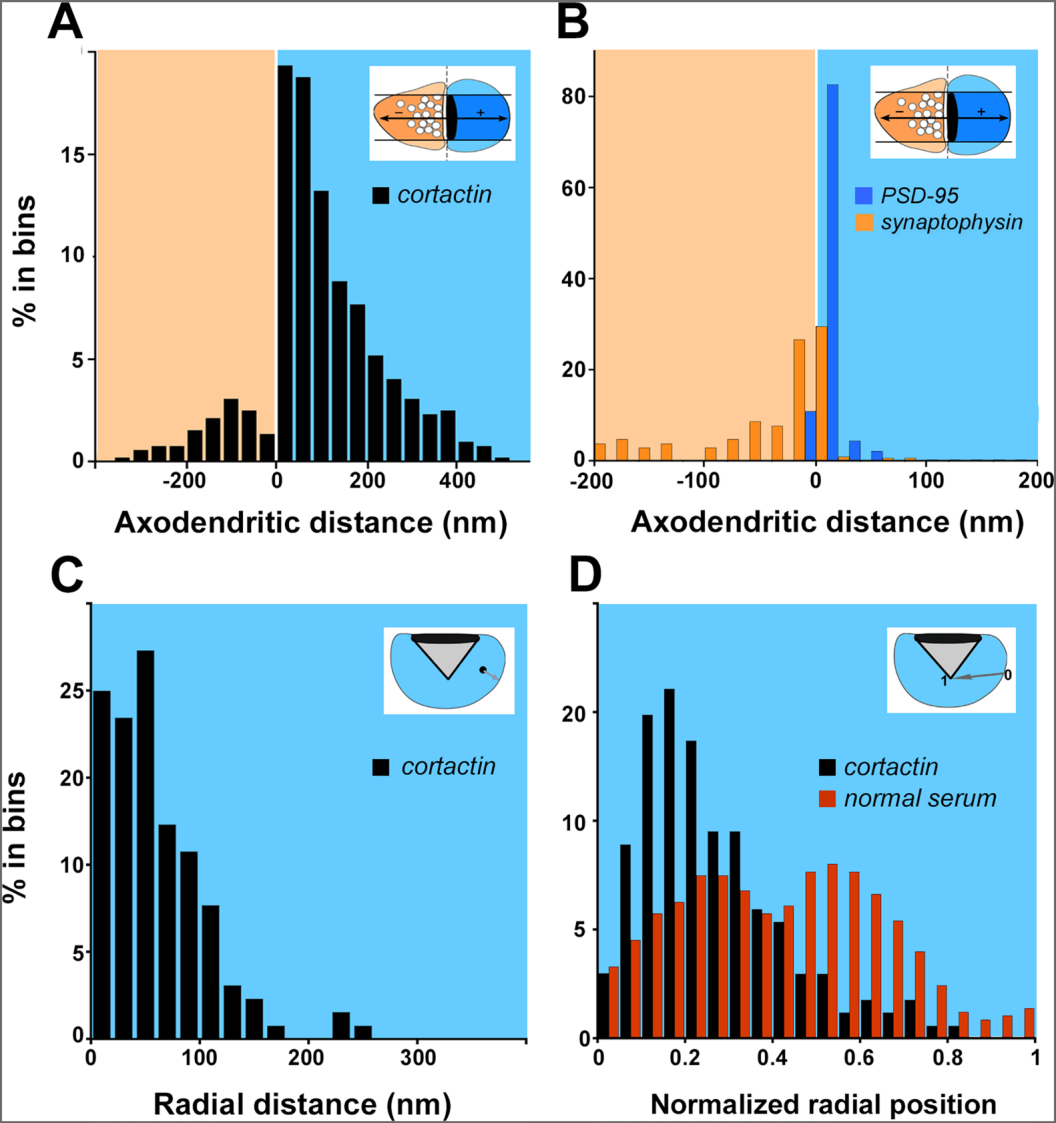
Distribution of cortactin, and other pre- and postsynaptic markers at PC-PF synapses. **(A**) Graph showing the axo-dendritic distribution of gold particles coding for cortactin. Cortactin accumulated at the synapse, extending into the cytoplasmic fringe of the PSD. (See insets for explanation: orange shaded area is presynaptic, while blue is postsynaptic) (**B)** Axo-dendritic distribution of gold particles coding for synaptophysin, and PSD-95. Synaptophysin concentrated presynaptically, while PSD-95 was highly restricted to the postsynapse. (See insets for explanation) (**C**) Radial distribution of particles coding for cortactin. Radial distances of gold particles coding for cortactin from the closest membrane of spine heads were shown in the graph as proportions. Most cortactin particles were found in the immediate vicinity of the non-synaptic membrane. (**D)** Normalized distribution of cortactin shows that cortactin, independent of spine size, is restricted to the submembrane domain while particles from samples containing normal rabbit serum as control were found to be distributed more evenly within the spinoplasm. Data are from 195 profiles for cortactin, 45 for synaptophysin, and 45 for PSD-95. The bin width was 35 nm for **(A)**, 20 nm for **(B,C**), and 0.05 normalized unit for **(D)**. In D data were smoothed with a three-point weighted running average.

PC spines were strongly labeled with immunogold (Fig. 4D1–4, asterisks). Particles coding for cortactin in spines were often associated with or in proximity to the spine membrane, including the postsynaptic density. To gauge the distribution of cortactin, we measured distances of gold particles coding for cortactin along the axo-dendritic axis (Fig. 5). Values along the axodendritic axis indicate position relative to the synaptic cleft. The axo-dendritic axis lies perpendicular to the axis of the synaptic cleft; negative values along the axo-dendritic axis indicate presynaptic distribution while positive values indicate postsynaptic distribution (small positive values indicate PSD localization; see Fig. 5B. PSD-95). Mean cortactin labelling was found 78 ± 11 nm from the PSD (n = 278 gold particles). Cortactin label was found to rapidly decline 150 nm away from the synaptic membrane, demonstrating its absence in the spine core domain. Additionally, we measured the average PSD thickness in the analyzed spine population (38 ± 1.5 nm, n = 102 spines, data not shown). We found that cortactin concentrates within or in the immediate vicinity of the PSD along the axodendritic axis, extending somewhat into the spinoplasm. We compared cortactin’s axodendritic distribution to that of synaptophysin (a presynaptic marker, Fig. 4E) and PSD-95 (a postsynaptic marker, Fig. 4F), confirming that cortactin has a significantly different distribution (-41 ± 8 nm for synaptophysin and 6 ± 1.5 nm for PSD-95 (n = 110 particles for both proteins), respectively; Fig. 5B).

Next, we investigated particle positions outside the PSD-defined synaptic membrane area. By measuring radial particle distances to the non-synaptic spine membrane, we can reveal whether cortactin is restricted to or accumulated in any spinoplasm domain. In this case, we did not consider particles lying within the wedge defined by the lateral edges of the PSD and the geometric center of the spine, in order to avoid bias because of the synaptic cortactin pool (see gray-shaded area in inset in Fig. 5C). We found that these particles were on average at 55 ± 3 nm from the non-synaptic membrane and they were virtually absent from the spine core beyond 200 nm from the membrane (Fig. 5C, n = 130 gold particles). Spine profiles had areas in the range of 0.01–0.2 µm^2^ and diameters of 300–600 nm (Table 2). Part of this apparent decline in labeling arises simply because many spine profiles had radii less than 200 nm. We computed a normalized position to correct for this with 0 corresponding to a particle at the plasma membrane, and 1.0 to a particle at the geometric center of the spine (see inset in Fig. 5D). This normalization allowed us to plot distribution independent of spine size.

**Figure 6:**
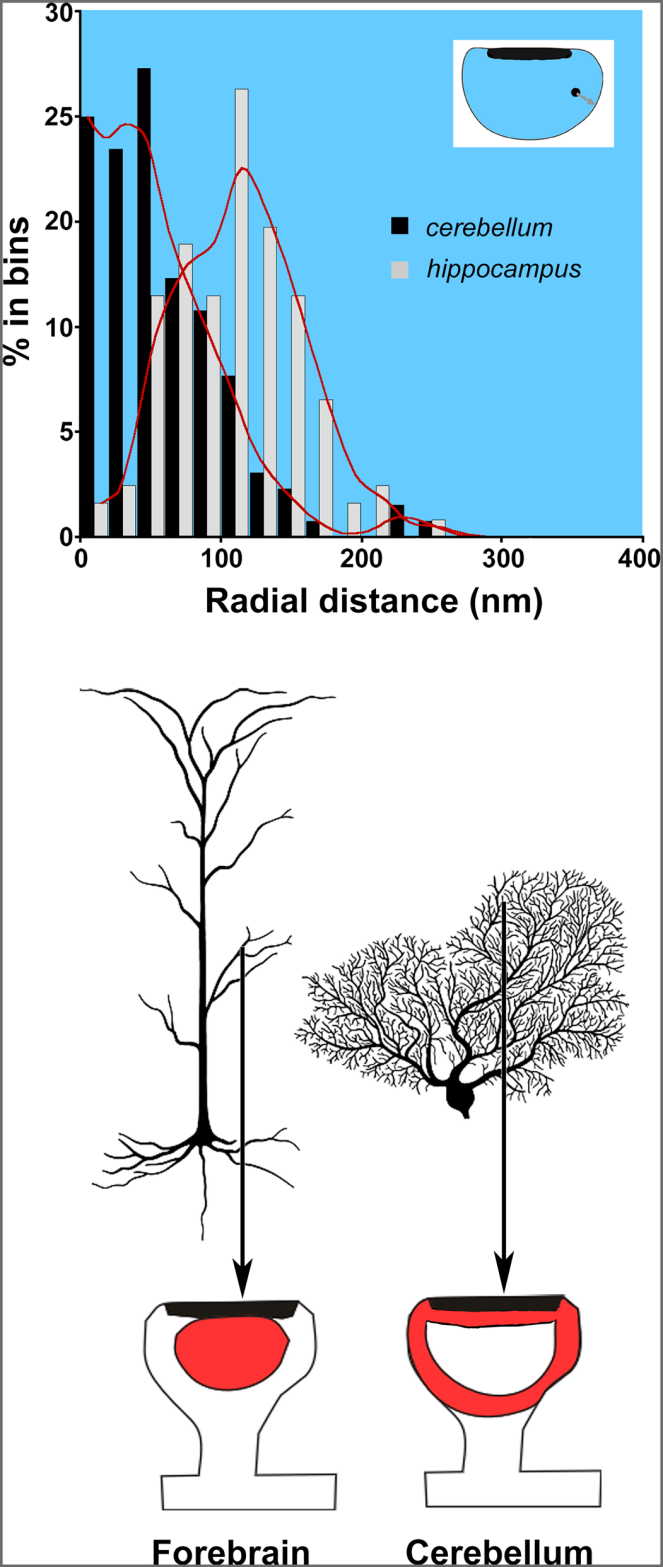
Different distribution of cortactin within forebrain, and cerebellar spines. Upper panel: graph showing the radial distribution of gold particles coding for cortactin in hippocampus (gray columns, data is from ^25^), and cerebellum (black columns). Cortactin accumulates in the core of hippocampal spines, while it resides in the shell in PCs (see inset for explanation of radial distributing particles; red lines are trend representations of the bar values as smoothed curves). Lower panel: schematic figure showing that cortactin in spines from apical dendrites of forebrain pyramidal neurons concentrates in a central, spinoplasmic core region, while PC spines have a shell-enriched cortactin pool.

Distance bins close to the center of the spine contain much less cytoplasmic area than those close to the membrane. Therefore, the number of particles close to the membrane would be higher than in the center of the spine even for uniformly distributed random points. We computed normalized labeling density to compensate for this reduction in bin area close to the center of the spine by dividing the number of counts in each bin by the normalized radius of the bin. After these corrections, the shell region was found to be highly concentrated in particles coding for cortactin with ~ 0.2 normalized units from the membrane (mean 0.26 ± 0.01 normalized units), whereas gold particles from the sections, where the primary antibody was replaced with normal rabbit serum, were found to be distributed evenly along the radial axis of the spine (mean 0.41 ± 0.01 normalized units; P < 0.001, Student’s t test; Fig. 5D). Importantly, the distribution of cortactin in PC spines-where it accumulates in the spine shell-differs from its distribution in hippocampal spines, where it resides in the spine core (Fig. 6).Taken together, cortactin prefers to concentrate in a submembrane domain within PC spines.

## Discussion

Since Ramón y Cajal’s drawings of Golgi-stained neurons that bear tiny protrusions, neuroscientists have debated the function of dendritic spines ^36^. Spines are a prominent feature of glutamatergic synapses, regardless of the brain region. Forebrain spines superficially resemble spines in the cerebellum, though PC spines are typically larger than cortical or hippocampal spines (showing larger head, and neck volume, PSD area, volume of presynaptic terminal etc.)^37–40^. However, there are also more significant differences between the two types of spines. For example, cerebellar spines contain abundant smooth endoplasmic reticulum (SER), loaded with IP_3_ receptors ^41,42^, whereas hippocampal spines contain only occasional endosomes, and rarely SER ^39^. Forebrain spines are highly dynamic during and after LTP, resulting in obvious changes in the morphology of thin, stubby, and mushroom spines ^43,44^, whereas cerebellar spines seem to be reluctant to change their shape during synaptic plasticity, maintain a mushroom-like spine profile at full developmental maturity ^3,34^. Moreover, forebrain and PC spines exhibit completely distinct developmental principals: the formation of PC spines is an intrinsically cell-autonomous phenomenon independent of a presynaptic partner ^45^, while the formation of hippocampal spines requires an active presynaptic axon terminal ^46^. These differences imply that the spines’ superficial morphological resemblance may be misleading.

The actin spinoskeleton is a highly dynamic branched network of filaments underlying most synaptic morphing observed during synaptic plasticity in the forebrain ^47^. Several proteins that regulate the organization of the actin cytoskeleton determine the shape of the spine ^47,48^. Actin filaments are directly involved in many synaptic functions, including anchoring and stabilizing synaptic glutamate receptors ^49^. Most excitatory forebrain spines have both NMDA- and AMPA-type glutamate receptors ^16,18,50,51^. However, glutamate receptors at the cerebellar PF-PC synapse are almost exclusively restricted to AMPA types^7–9^ (although the CF-PC synapses may express NMDA receptors). Therefore, the synapse-receptor-spinoskeleton relationship is likely different in the two brain regions, as NMDA receptors are directly linked to actin through α-actinin, whereas AMPA receptors bind to actin filaments more indirectly ^17^. At PC-PF synapses, major players include mGluR1, whose activation triggers downstream signaling pathways that indirectly promote the reorganization of the actin cytoskeleton ^52,53^. These dramatic differences in receptor mechanisms raise the possibility of corresponding differences in regulation of the actin cytoskeleton. Our findings that cortactin is organized differently in cerebellar spines as compared to forebrain spines warrant further molecular, and functional studies.

Cortactin may also help to mediate various synaptic defects through these different structural changes. Cortactin is known to interact with the Shank family of postsynaptic scaffolding proteins through its SH3-domain, modulating the interaction between the synapse and the spinoskeleton ^54^. Shank3 is the predominant isoform at forebrain spine synapses, whereas cerebellar spine synapses express mainly Shank2 ^55,56^. Loss of Shank3 results in a notable change in forebrain spines and synapses (consistent with its role in autism) ^56^. However, loss of Shank2 from the cerebellum has no obvious impact on the morphology of PC dendritic spines or PSDs ^55^. Our finding that cortactin has an entirely different organization in cerebellar PC spines compared to forebrain spines might explain these differences in spine pathology.

Through its interaction with the brain-exclusive cortactin-binding protein 2 (CTTNBP2 also known as CortBP2 or CBP90), cortactin may also be involved in the regulation of neuronal spine density, as CTTNBP2 is pivotal in spinogenesis^57^. The interaction between these two proteins is also important in defining the morphological phenotype of spine-heads. Our results suggest a mechanistic explanation for how cortactin may act differently in forebrain vs. cerebellum: its different distribution within spines may underlie the distinct morphological and plastic features seen in these two brain regions.

## Supplementary Information

Supplementary Figure S1.

## Data Availability

The data supporting our findings in this study are available from the corresponding author (BR) upon reasonable request.
