# Supplementary material for: Distribution of cortactin in cerebellar Purkinje cell spines
Source: Sci Rep. 2021 Jan 14;11:1375. doi: 10.1038/s41598-020-80469-w (PMC7809465; doi:10.1038/s41598-020-80469-w)
Supplement: Supplementary file 1 — Supplementary Figure S1. [file 41598_2020_80469_MOESM1_ESM.pdf]

Distribution of cortactin in cerebellar Purkinje cell spines

Lilla E. Szabó[#], G. Mark Marcello[#], Miklós Süth, Péter Sótonyi, Bence Rácz*

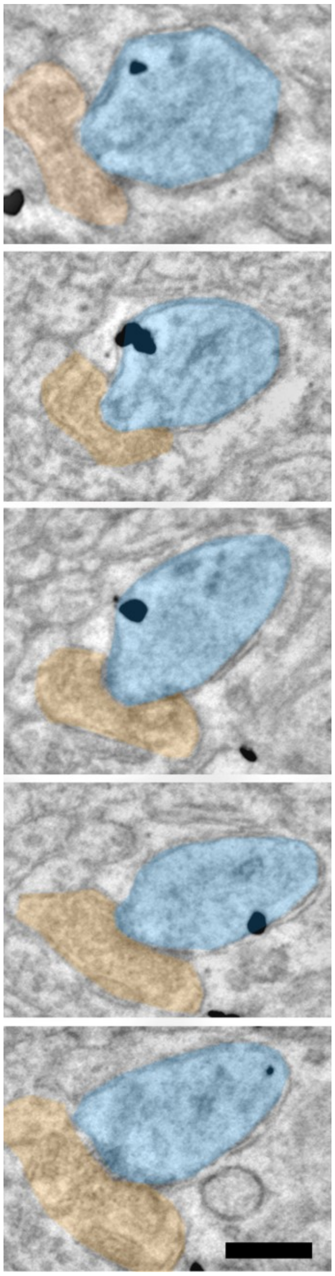

Supplementary Figure S1. Serial-section electronmicroscopy of a Purkinje cell-parallel fiber synapse. Immunogold label in PC spine profiles are coding for cortactin. Dendritic spine of PC (blue pseudo-color) and PF terminal (orange pseudo-color). Cortactin-coding immunogold particles can be seen in five consecutive sections associated with submembrane spinoplasm in the periphery of spines. Scale bar 250nm.
